# Argan Oil: Chemical Composition, Extraction Process, and Quality Control

**DOI:** 10.3389/fnut.2021.804587

**Published:** 2022-02-03

**Authors:** Said Gharby, Zoubida Charrouf

**Affiliations:** ^1^Laboratory Biotechnology, Materials and Environment, Department of Chemistry and Physics, Polydisciplinary Faculty of Taroudant, Ibn Zohr University, Taroudant, Morocco; ^2^Laboratory of Plant Chemistry and Organic and Bioorganic Synthesis, Department of Chemistry, Faculty of Sciences, Mohammed V University, Rabat, Morocco

**Keywords:** argan tree, argan oil, chemical composition, quality, oxidation, extraction

## Abstract

Argan oil is considered a relatively international product exported from Morocco, although different companies in Europe and North America distribute argan oil around the globe. Argan oil is non-refined vegetable oil, of the more well-known “virgin oil” type, is produced from the argan tree [*Argania spinosa* (L.) Skeels]. The argan tree is deemed to be an important forest species from both social and economic standpoints. Argan oil has rapidly emerged as an important product able to bring more income to the local population. In addition, it also has important environmental implications, owing to its ability to stand against desert progression. Currently, argan oil is mainly produced by women's cooperatives in Morocco using a semi-industrial mechanical extraction process. This allows the production of high-quality argan oil. Depending on the method used to prepare argan kernels, two types of argan oil can be obtained: food or cosmetic grade. Cosmetic argan oil is prepared from unroasted kernels, whereas food argan oil is achieved by cold pressing kernels roasted for a few minutes. Previously, the same food argan oil was prepared exclusively by women according to a laborious ancestral process. Extraction technology has been evolved to obtain high-quality argan oil at a large scale. The extraction process and several accompanying parameters can influence the quality, stability, and purity of argan oil. In view of this, the present review discusses different aspects related to argan oil chemical composition along with its nutritional and cosmetic values. Similarly, it details different processes used to prepare argan oil, as well as its quality control, oxidative stability, and authenticity assessment.

## Arganeraie and Argan Tree

Through the ages, the Amazigh population, the indigenous inhabitants of North Africa, has inhabited the arganeraie area of Morocco and has developed a lifestyle that depends on the argan tree. In Southwest Morocco, there was a noticeable degradation of argan forest from about 1.5 million ha to only an estimated 800,000 ha by the year 2000 (1, 2). The decline is anthropogenic but is also due to drought. As a consequence, Unesco declared the Moroccan argan forest a biosphere reserve in 1998 (1, 3). Since then, a comprehensive program aiming at saving the argan tree in a sustainable way has been underway in Morocco (4). Among its significant results is the scientific confirmation of traditional medicinal values virtues arising mostly from the main product, which is argan oil. Since then, argan oil is gaining significant value worldwide with increasing demand from year to year. In addition, in 2021, the United Nations Organization (UNO) recognized the importance of the argan tree by adopting May 10 as its international day.

Argan tree is an endemic plant found principally in Morocco (5), and it extends as far as the Hamada of Tindouf in Algeria (6). In addition, this tree has also been introduced as a cultivated species in the deserts of South Africa, Spain, Kuwait, Mexico, Tunisia, and Israel, among other countries of the world (7, 8).

Botanically, the argan tree is the only species of the *Sapotaceae* family to live above the tropics (9). It is a slow growing and thorny tree, well-adapted to the drylands of south-western Morocco (9). In its biotope, it plays an important ecological role including a deep root system that protects the soil from water and wind erosion, while its shade maintains soil fertility by preserving moisture (10, 11). In addition, the argan tree has multiple uses. Its leaves, fruits, and oil are a source of nutrients and bioactive compounds with nutritional, cosmetic, and economic benefits (12, 13). In this chapter, the chemical composition of argan oil along with its nutritional and cosmetic benefits will be discussed. We will detail different processes used to process argan oil, quality control, and assessment of oxidative stability.

## Importance of Argan Oil as a Food and Cosmetic Product

It is widely evidenced that argan oil is an important food. It is prepared from roasted argan kernels, whose pressing can be performed using traditional and modern techniques (14–16). In both cases, the obtained argan oil has a golden color and a unique hazelnut taste (17). Food argan oil is sweet with a nutty flavor and it is known as one of the most nutritional oils in the world. This explains the consumer demand as well as the incorporation of argan oil into other several varieties of foods. Traditionally, argan oil used as a food is eaten with bread. In addition, it makes a great finishing flavor when drizzled over grilled salads, fish, tajin, and couscous. Moreover, food argan oil is also the main ingredient in Amlou, a paste similar to peanut butter obtained by grinding roasted almonds with honey and argan oil (Guillaume and Charrouf, 2013). A few years ago, argan oil became highly valued by major chefs in Europe. Food argan is the main source of fat used for food preparation in the Amazigh diet and, therefore, it is deemed to be symbolic of this cultural continuity (18–20).

Several scientific studies devoted to the development of argan oil for over 30 years have shown various promising nutritional characteristics of this product in human health (21).

There are two kinds of argan oils in the market, namely, food argan oil and beauty (cosmetic) use (*name INCI: Argania spinosa kernel oil*) (21). This is a cold pressed oil, however, it is obtained from unroasted argan kernels (15, 22). Cosmetic argan oil is intended to be applied directly to the skin or hair, or to be an ingredient in other cosmetic preparations (21, 23, 24). Traditionally, cosmetic argan oil has been claimed to cure all kinds of skin anomalies with particular effectiveness for juvenile acne and chickenpox. Additionally, it is also claimed to reduce the formation of wrinkles. Argan oil is generally used in cosmetology as a moisturizer as well as to cure devitalized skin. Added to shampoos, the oil penetrates the hair axis and damaged hair follicles, giving hair a fuller and shinier look. All these skin healing properties justify the wide use of argan oil in dermatological and cosmetic formulations (25).

Nutritional and cosmetic properties of either argan oil devoted to direct consumption or cosmetic end uses are the result of its unique chemical composition. Argan oil is rich in unsaturated fatty acid and bioactive molecules, such as polyphenols, tocopherols, squalene, xanthophyll, CoQ10, and sterols (7, 26).

## Argan Oil Composition

**Figure 1:**
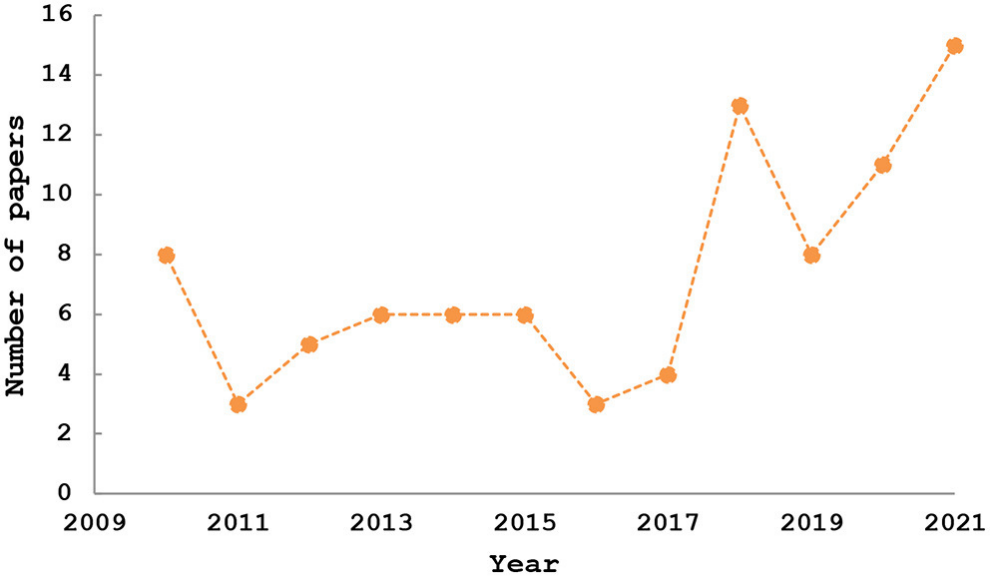
Publication trends of argan oil composition (Based on data retrieved from Scopus database). Copyright © 2022 Elsevier B.V. All rights reserved. Scopus® is a registered trademark of Elsevier B.V.

A complete and updated analysis of the composition of olive oil reported in the literature is presented in Figure 1. In fact, on November 27, 2021, the Scopus database was chosen for the search. The search string: (“argan oil^*^” AND “composition^*^”) was utilized to extract bibliometric information from the Scopus online database.

The obtained results (Figure 1) showed that argan oil composition is well*-*documented.

Indeed, 86 publications were recovered through the literature search within the range of years from 2010 to 2021, 34 of them which represent about 40% are published in the interval of years from 2015 to 2021.

### Physicochemical Parameters

Parameters systematically used to measure physicochemical properties of argan oil include density, refractive index, iodine value, saponification value, and unsaponifiable matter. In vegetable oils including argan oil, the density and refraction index depend on the temperature (27). Table 1 summarizes the ranges of the main physicochemical parameters of argan oil. The refractive index at 20°C varies from 1.463 to 1.472. At the same temperature, its density relative to water is between 0.906 and 0.919 (28). The iodine value is a measure of the total number of double bonds present in the oil sample (29). Argan oil shows an iodine value ranging from 91 to 110 mg/100 g (28, 30) (Table 1). This value is lower than that of sunflower oil (130 g I_2_/100 g) and soybean seed oil [134.5 g (I_2_)/100 g] but higher than that of olive oil [75–94 g (I_2_)/100 g] (31, 32). High iodine-value is correlated to a greater number of double bonds and reduced oxidative stability (32, 33). The saponification value is a measure of the average fatty acid chain lengths. The oil with shorter fatty acid has a higher saponification value. The saponification value of argan oil according to SNIMA norm is 189.0–199.1 mg (KOH)/g. It is noteworthy that a high saponification value indicates high triglycerides content. This aspect is very appreciated in cosmetology (32).

**Table 1:** Physicochemical parameters of cosmetic and edible argan oil.

**Physicochemical properties**	**Norm (28)**
Density (20°C)	0.906–0.919
Refraction index (20°C)	1.463–1.472
Saponification value (mgKOH/g)	189.0–199.1
Iodine value [g (I_2_)/100 g]	91.0–110.0
Unsaponifiable (g/100 g)	≤ 1.1

### Fatty Acid, Phytosterol, Tocopherol, and Volatile Compounds Composition

The most important components in vegetable oils are fatty acids (Table 2). It is noteworthy that the characteristics, stability, and nutritive value of a given vegetable oil depend strongly upon the fatty acid composition (34, 35). In this regard, argan oil is composed of about 80% of unsaturated fatty acids and only approximately around 19 g/100 g saturated fatty acids (Figure 1). The nutritional benefits of unsaturated fatty acids are widely documented (36, 37). Oleic acid (43–49 g/100 g) and linoleic acid (29–37 g/100 g) are the major unsaturated fatty acids found in argan oil (4, 20, 38). Linolenic acid, a very oxidizable molecule, is also present in very low concentrations usually <0.3 g/100 g (39). This small content of linolenic acid can be used to detect the adulteration of argan oil with other vegetable oils rich in linolenic acids, such as soybean and rapeseed oils (39). Argan oil also contains two primary saturated fatty acids namely stearic acid (3.14–3.28 g/100 g) and palmitic acid (11.70–11.75 g/100 g) (1, 40). Other fatty acids, such as myristic acid (C14:0), palmitoleic acid (C16:1), arachidic acid (C20:0), and behenic acid (C22:0) are found only in relatively lower quantities (41).

**Table 2:** The fatty acids, phytosterols, and tocopherols composition of argan oil.

**Fatty acid (g/100 g)**	**Norm (28)**	**Tocopherol**	**Norm (28)**
Myristic acid (C14:0)	≤ 0.2	α-Tocopherol (g/100 g)	2.4–6.5
Palmitic acid (C16:0)	11.5–15	β-Tocopherol (g/100 g)	0.1–0.3
Stearic acid (C18:0)	4.3–7.2	γ-Tocopherol (g/100 g)	81–92
Arachidic acid (C20:0)	≤ 0.5	δ-Tocopherol (g/100 g)	6.2–12.8
Behenic acid (C22:0)	≤ 0.2	Total tocopherol (mg/100 g)	60–90
Σ SFA	15.8–23.1	Phytosterol (g/100 g)	Norm
Palmitoleic acid (C16:1)	≤ 0.2	Cholesterol	≤ 0.4%
Oleic acid (C18:1)	43.0–49.1	Campesterol	≤ 0.4%
Eicosenoic acid (C20:1)	≤ 0.5	Δ-7-Avenasterol	4.0–7.0
Σ MUFA	43–49.8	Schotenol	44.0–49.0
Linoleic acid (C18:2)	29.3–36.0	Stigmasta-8-22-dièn-3β-ol	3.2–5.7
Linolenic acid (C18:3)	≤ 0.3	Spinasterol	34.0–44.0
Σ PUFA	29–36.3	Total sterol (mg/100 g)	≤ 220

*SFA, saturated fatty acids, MUFA, monounsaturated fatty acids, PUFA, polyunsaturated fatty acids*.

**Figure 2:**
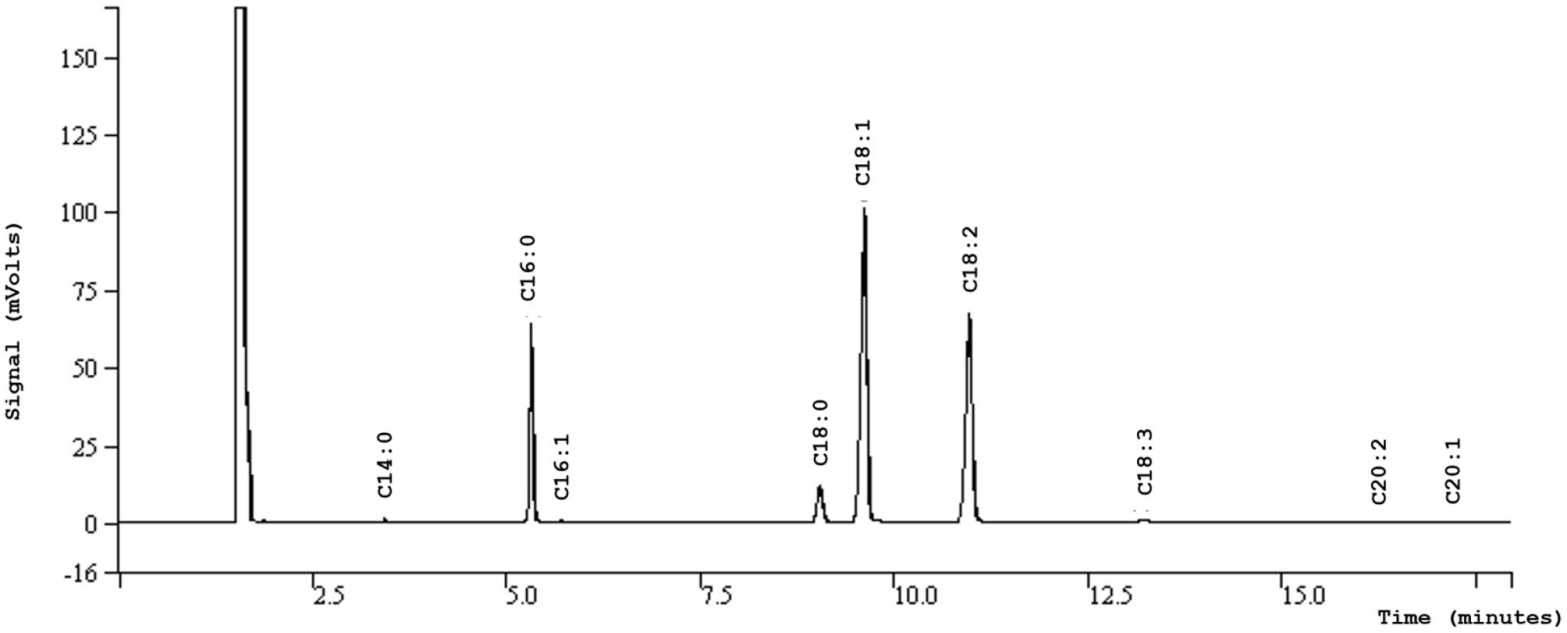
GC chromatogram of argan oil fatty acids.

**Figure 3:**
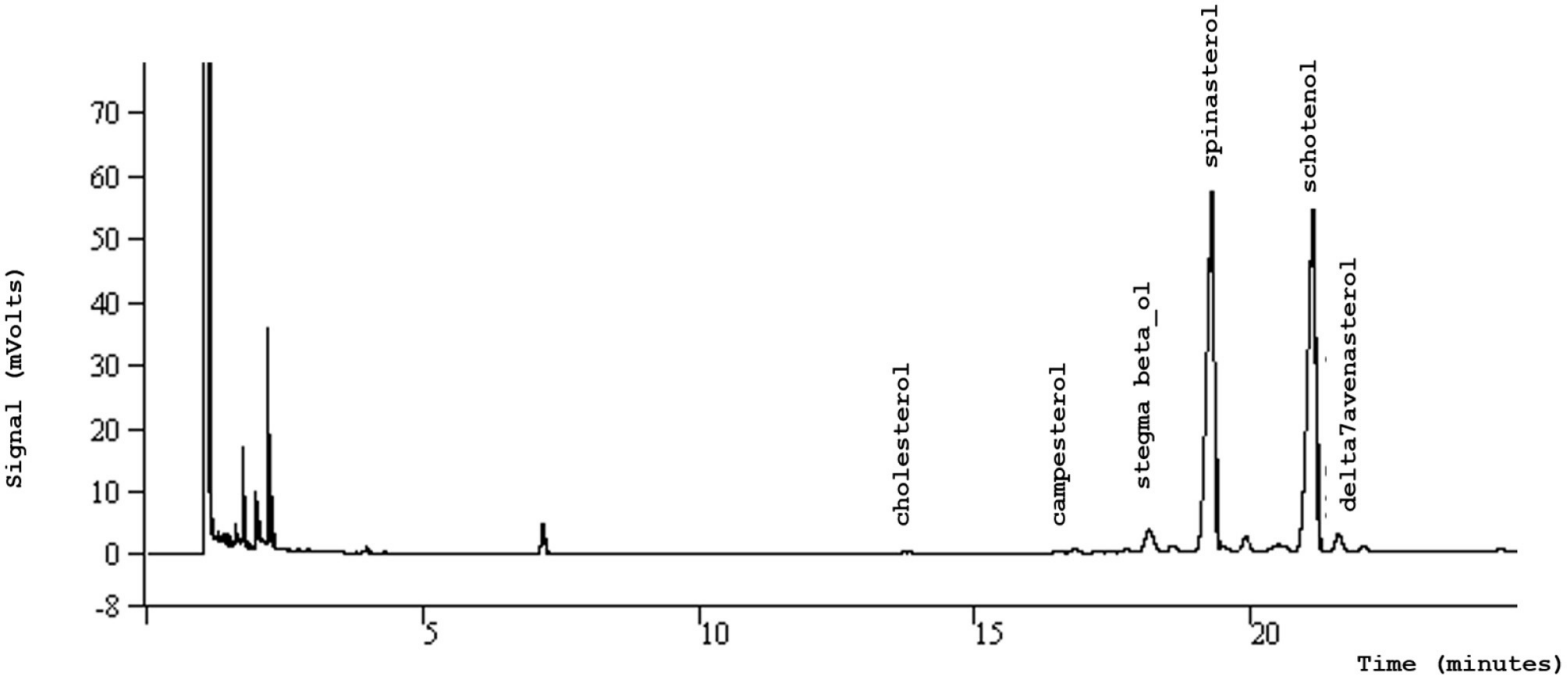
GC chromatogram of sterols of argan oil.

Sterols, otherwise referred to as phytosterols, are the second class of compounds found in argan oil after fatty acids (Figure 2). The content of phytosterols in argan oil may be up to 220 mg/100 g (28, 30). They are usually used to prove authenticity since they can be considered as a fingerprint of argan oil (42). In addition, sterols are important molecules endowed with potent biological properties and their determination is of major interest due to their antioxidant activity and beneficial impact on health (43–45). Spinasterol (34.0–44 g/100 g) and Schotenol (44.0–49 g/100 g) are the major argan oil sterols followed by Δ7-Avenasterol (4.0–7.0 g/100 g) and stigmasta-8-22-dièn-3β-ol (3.2–5.7 g/100 g) (46) (Table 2; Figure 3). Campesterol is found at a very low level but this is interesting for argan oil authenticity assessment (47, 48). This is also rich in squalene, with content much higher than that found in sunflower oil, but is lower than that of the olive oil (49, 50).

In addition to the fatty acid composition and phytosterol profile, the composition of tocopherol compounds is an important characteristic of argan oil (51). The tocopherol composition is closely related to stability, cosmetic, and nutritional proprieties (39, 52) and can be used for detecting adulterations in argan oil (53). Argan oil is very rich in tocopherols (15, 51). The total tocopherol content is between 60 and 90 mg/100 g. γ-tocopherol is the main tocopherol found in argan oil (between 81 and 92 g/100 g), followed by δ-tocopherol (6.2–12.8 g/100 g), α-tocopherol (2.4–6.5 g/100 g), and β-tocopherol (0.1–0.3 g/100 g) (28, 46). In addition, it contains other bioactive compounds of important antioxidant power, such as polyphenols (54), coenzyme Q10, and melatonin (26).

A large number of different classes of chemical compounds, such as hydrocarbons, ketones, furans, and aldehydes, participate in the final aroma of argan oil (55). The volatile fraction of virgin argan oils has been reported to have approximately eight major families: acids, alcohols, aldehydes, ketones, esters, terpenes, N-heterocycles, and furans (55). However, argan oil obtained from goat-peeled fruit possesses additional volatile compounds (56). The volatile compounds of cold pressed argan oil are strongly dependent on the roasting process that is particularly applied to food-grade argan oil. In fact, during the process of roasting, an aroma is developed, which is preserved in the oil during the process of extraction. The principal ways of formation of the flavor during the roasting of argan kernels are the oil oxidation and the Maillard reaction (19). Indeed, the volatile profile of the oil extracted from unroasted kernels is dominated by the presence of oil oxidation products, such as hexanal, pentanal, and 2-pentyl-furan, and by N-heterocycles, such as 1-methyl-1-pyrrole, 2,6-dimethyl pyrazine, and other pyrazines, alcohols (1-hexanol, 2,3-butanediol, 2-methyl-1-propanol), ketones (acetoin), and acetic acid (55, 57). However, the volatile content of food grade oil is significantly marked by an increased level of products of the Maillard reaction, such as the Strecker aldehydes 2-methyl propanal, 2- and 3-methyl butanal, and the N-heterocycles 1-methyl-1-pyrrole, methyl pyrazine, 2,6-dimethyl pyrazine, 2-ethyl-5-methyl pyrazine, trimethyl pyrazine, 3-ethyl-2,5-dimethyl pyrazine, and furfural. Matthäus et al. (19) have previously demonstrated that hexanal can be identified as an oxidation indicator in both untreated and roasted argan oils. Furthermore, El Monfalouti et al. (55) investigated the impact of roasting time on the concentration of volatiles in the extracted oil, emphasizing that almost all roasting-related volatiles commenced being generated after 15–25 min of roasting. In another study, Gracka et al. (57) characterized the major odors, using gas chromatography olfactometric analysis, and the sensory properties of raw and roasted kernel oils. The two oils showed increased oily and rancid olfactive notes at the end of an accelerated storage experiment, whereas certain aldehydes were recognized as potential indicators of oxidation during storage (57).

## Argan Oil Extraction

**Figure 4:**
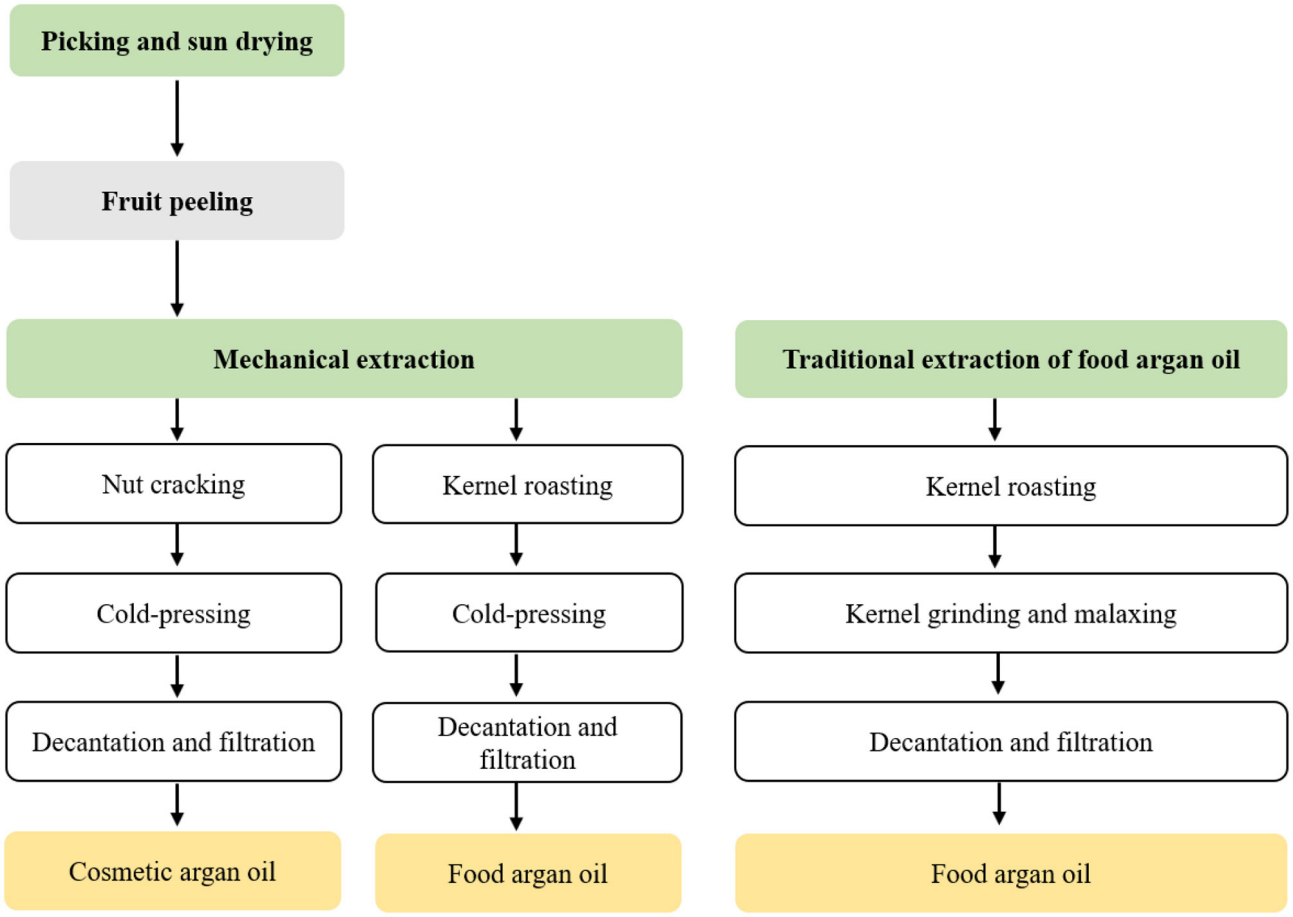
Scheme of argan oil extraction by the pressing method.

For a long time ago, argan oil has been prepared exclusively by Amazigh women according to a laborious ancestral traditional extraction method (40, 58). The traditional process of extraction, transmitted from mother to daughter, includes seven steps (Figure 4): (1) fruit picking: from May to August, ripe fruits are collected from the argan forest. The fruits are collected manually and sun-dried for a few weeks. Harhar et al. (59) showed that a 2-week fruit drying time is optimum to prepare the high quality of argan oil. (2) Fruit peeling: following the drying period, their dried peel is manually removed, resulting in “argan nuts”; (3) nut cracking: the argan nuts are broken open, kernels selected, and collected. (4) Kernel roasting: the kernels are then gently roasted in clay plates for a few minutes. The amount of time depends on each woman who evaluates it based on the color and the odor of the kernels. An average of 30–40 min is necessary to roast 1 kg of kernels. Roasting time strongly influences the final oil taste. A burning taste results from overheating of the material and needs to be avoided. This step is necessary to get limpid oil having reproducible color and odor characteristics. (5) The fifth step consists of kernel grinding: the roasted kernels are crushed using a millstone. This artisanal process is composed of two stones: a bedstone and a cone-shaped rotating piece largely pierced in its center through which the kernels are introduced. (6) In the sixth step, i.e., malaxing: the resultant oily dough is hand-malaxed for several minutes, while small quantities of water are added. As the dough slowly gets solid, it releases an emulsion from which argan oil is decanted. (7) Oil extraction: the final stage is traditional oil extraction. This process is very laborious. Indeed, for a single person, 24 h of work is needed to extract about 1 L of oil from 50 kg of fruits. The solid residue remaining after the maximum quantity of oil has been collected may still contain up to 25% of oil. In addition, traditional extraction is frequently achieved in unsatisfactory sanitary conditions, particularly, in terms of bacteriological safety, traceability, and oxidative stability (17). As a solution, a change was made to increase argan oil quality and traceability by improving its extraction technology.

The introduction of a mechanical technique by several industrial units and women's cooperatives improved not only the quality certified of oil but also its yield (41). In fact, the oil preparation process was only slightly modified: the fruits are peeled mechanically, and the oil is obtained by press extraction. In the advancement of this process, the use of water for extraction has been eliminated and the roasting step has been improved. This process has two advantages. First, argan oil can join olive oil as a “virgin extra argan oil” and, second, the new technology preserves all the known benefits of argan oil. As of this writing, argan oil is still being prepared in some sectors of Morocco using mechanical presses, however, in some industrial units and cooperatives, highly efficient, hydraulic presses have replaced endless crews (4). With this process (mechanical or hydraulic process), two types of oil can be obtained: food or cosmetic grade (48). Food argan oil is achieved by cold pressing kernels roasted for a few minutes, whereas cosmetic oil is prepared from unroasted kernels (14, 58). Food argan oil extraction follows six steps: fruit collection, sun-drying, dehulling, nut breaking (or kernel collection), kernel roasting, and cold-pressing. Oil decantation and filtration are also added to remove large amounts of waxes and gums. Skipping the roasting step leads to the production of cosmetic oil. Food argan oil is copper-colored whereas cosmetic argan oil is gold-colored. Each processing step dramatically influences the resulting oil in terms of quantity and quality. The importance of the extraction method on the oil quality is already particularly well-documented (19, 41). The effects of fruit quality and drying-time on argan oil quality have also been evaluated (19, 22, 41, 59). Concerning the roasting stage, the impact of its duration and the resulting volatile compounds production are well-documented (22). Besides, food argan has a hazelnut-like taste, mostly resulting from the presence of volatile compounds formed during kernel roasting. Volatile compounds, produced *via* oxidation, and/or Maillard reaction, actively participate in the preservation of food oil (19, 60, 61).

## Quality Control of Argan Oil

Argan oil is subject to numerous analytical and sensory controls to assess its overall quality. These analyses specify the freshness of the oil concerning hydrolytic and oxidative alterations to ensure the conformity of products to their labels. For example, “extra virgin argan oil” by simple analyses [free fatty acids, peroxide value, and specific extinction (E270 and E232)] and/or purity blending with other oils and contaminants. These criteria require detailed analyses (triglycerides contents, fatty acids, sterols, tocopherols, etc.). In addition, organoleptic characteristics (taste, odor, and color) also need to be taken into consideration.

As with other vegetable oils, the oxidation of argan oil leads to natural phenomena alteration (60, 62). This can be controlled from fruit harvest until the storage of the oil. Because of the possibility of oxidation, physicochemical criteria, such as acidity, peroxide value, and extinction specific at wavelength 270 (E270), have been selected as the backbone of argan oil quality determination by the Moroccan Standard (08.5.090). Acidity of argan oil is classified in four grades: extra-virgin (acidity < 0.8 g/100 g), fine-virgin (0.8 < Acidity < 1.5 g/100 g), ordinary virgin 1.5 < Acidity < 2.5 g/100 g), and lampante virgin argan oil (acidity > 2.5) (28).

The variability of the extra-virgin argan oil acid value as a function of various parameters has been studied (41, 60). In addition, seed origin and the technology associated with extraction are parameters possibly modifying the argan oil acidity (41, 60, 63). The oxidative state of oil can be examined from peroxide value and specific extinction coefficient (E232), which indicates the presence of primary oxidation products (20, 64). The peroxide value of extra-virgin argan oil must be lower than 15 mEq O_2_/kg (65) and specific extinction E232 is not yet been set in The Moroccan standard (28). Nevertheless, Kharbach et al. (66) have considered that the specific extinction E232 should not exceed 2.52 for extra virgin argan oil. The other two important indices used to evaluate the secondary oxidation products are p-anisidine value and specific extinction E270 (20, 64). The Moroccan Standard has fixed 0.25 as a limit value for extra virgin argan oil. In addition to the oxidation and acidity concerns, the quantification of major compounds, such as fatty acids, and minor compounds, such as sterols, polyphenols, tocopherols, and minerals elements, are also important considerations for the purity of argan oil.

Finally, identifying contaminants is one of the multiple checks that must be conducted on oils. Vegetable oils have fixed limit values for heavy metals, some mycotoxins, aromatic hydrocarbons polycyclics (PAHs), phthalates, and pesticides. Although the physicochemical characterization of argan oil is an essential step, it is not sufficient (60). To satisfy the consumer, organoleptic characteristics (taste, smell, color, etc.) must be taken into consideration. This is particularly true for food argan oil. An organoleptic analysis is an essential criterion for successful food marketing and an integral part of evaluating food argan oil.

## Argan Oil Oxidative Stability

Oil oxidation is a key factor responsible for oil quality during storage time. The loss of oil nutritional quality and the rise of undesirable off-flavors. This makes foods containing lipids less acceptable to consumers. Moreover, during the oxidation process, some toxic compounds, such as reactive carbonyl compounds can generate advanced lipid peroxidation end products which are potentially harmful to human health (67). Argan oil oxidation depends on oil fatty acid composition, the presence of endogenous antioxidants, and external factors, such as high temperatures and high oxygen availability (62). It is an undesirable set of chemical reactions that can be perceived, at the initial stage, as a deterioration of both taste and smell (rancidity). The oxidation of oil begins with radical reactions on unsaturated fatty acids and involves multiple stages, resulting in various decomposition products. Most of them are peroxides (primary oxidation products), while alcohols and volatile compounds (aldehydes and ketones) constitute secondary oxidation products (29).

In this context, argan oil oxidative stability is extensively documented (19, 34, 51, 53, 60, 68, 69). Several techniques and studies have been used to estimate the shelf-life of argan oil, such as Rancimat induction time, under accelerated conditions at 60°C as well as estimating its shelf-life for 2 years at 25°C (protected or exposed to sunlight) (60, 61, 70). Despite its high unsaturated fatty acid content, food argan oil prepared by press cold extraction has been proven to display a very good shelf-life and high preservation characteristics (60). For instance, at 110°C, food argan oil prepared by press cold extraction present an average Rancimat induction period of 31 h, whereas it is only 13 h for cosmetic argan oil. At high storage temperature (60°C) for 35 days, argan oil from roasted kernels prepared by press cold extraction shows high oxidative stability (19). Previous works (60, 69, 70) have estimated argan oil shelf-life for a duration of 2 years at 25°C in protected conditions or exposed to sunlight. The obtained results indicated that the food argan oil prepared by press cold extraction, for a storage period of 2 years showed that the best stability is obtained in the shelter of light and at 25°C. However, under the same conditions, cosmetic argan oil can only be preserved for 1 year (46, 69). Kharbach et al. (71) have studied the shelf-life of food and cosmetic argan oils based on chemical properties or Fourier Transform Infrared (FTIR) fingerprints and chemometrics. Since cosmetic and food argan oil differs only by kernels roasting and the life span of food argan oil is longer than that of cosmetic oil. More precisely, the moisture content in the food argan oil is lower, its phospholipid content (70) and the formation of antioxidant active compounds during the roasting process (e.g., could be responsible for this stability) is higher.

## Determination of Virgin Argan Oil Adulteration

**Figure 5:**
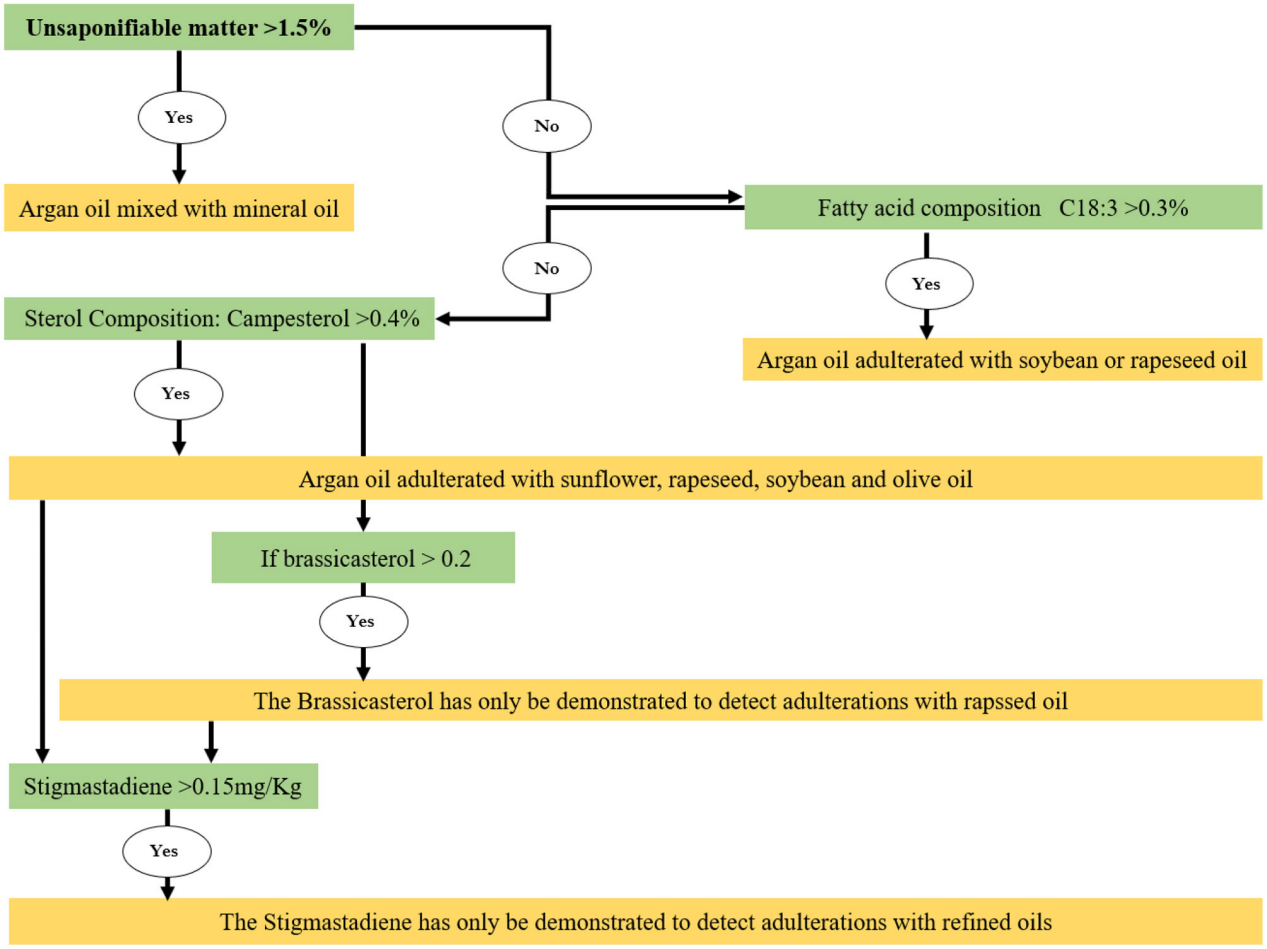
Scheme of the main analytical methods for determination of argan oil adulteration by vegetable oils.

Detection of food adulteration is of great importance to ensure product quality and consumer protection. The multi-stage extraction process and the superior value over other oils explain the high price of argan oil. For these reasons, this oil has been called “the most expensive vegetable oil in the world.” Unfortunately, this position increases the risk of adulteration using other lower-cost oil. To reduce this risk, several official analytical methods have been developed and proposed to detect adulteration in both cosmetic and food argan oil (Figure 5). The different methods of dosage are reviewed: unsaponifiable matter fatty acid, sterol, tocopherol, triglycerides, trace elements, and polyphenol spectrometric. These methods are widely applied to quality research on vegetable oils, such as olive oil. Adulteration of argan oil with cheaper oils rich in linolenic acid (rapeseed and soybean oils) can be easily and quickly detected by the determination of the fatty acid composition using gas chromatographic since linoleic acid represents only <0.3% in argan oil. Indeed, Miklavcic et al. (72) have investigated the authenticity of cosmetic argan oil using fatty acid composition. With the same technique, other authors reported that adulterations of this oil could be detected by the analysis of campesterol of phytosterol composition. Indeed, campesterol, a sterol found in vegetable oils, is present only in traces (0.4%) in the total sterols content of argan oil. This low level of campesterol in argan oil can be used to detect adulteration of argan oil with other oils rich in campesterol, such as olive, soybean, rapeseed, and sunflower oils (42). This analysis determines argan oil purity up to 98% (4). In addition, quantification of tocopherols (53, 73), hydrocarbons (74), ferulic acid (75), triglyceride (76), and stigmasta-3,5-diene has been reported. Dosage of trace elements is suggested as another analytical method for the assessment of argan oil adulteration (13, 14, 77). Other authors have used the content of unsaponifiable matter to detect the adulteration of argan oil by mineral oil (4). Recently, El Orche (78) has used three fingerprint analytical approaches based on spectroscopic sensors and chemometrics for the detection and quantification of argan oil adulteration (78).

## Conclusion

In this chapter, various aspects related to chemistry, different processes used for argan oil preparation, quality control, oxidative stability, and authenticity assessment of argan oil are summarized in light of published literature. A few years ago, both types of argan oil (cosmetic or food) have found their place in the highly competitive international oil market. This is the result of its unique chemical composition associated with its nutritional and cosmetic properties as well as with scientific studies validating its claimed or empirically observed properties. With such an approach, the commercial success of argan oil has been achieved globally. This success is a very positive sign for the preservation of argan oil.

## Author Contributions

SG: resources, data acquisition, methodology, and writing—review and editing. ZC: supervision and writing—review and editing. Both authors contributed to the article and approved the submitted version.

## Conflict of Interest

The authors declare that the research was conducted in the absence of any commercial or financial relationships that could be construed as a potential conflict of interest.

## Publisher's Note

All claims expressed in this article are solely those of the authors and do not necessarily represent those of their affiliated organizations, or those of the publisher, the editors and the reviewers. Any product that may be evaluated in this article, or claim that may be made by its manufacturer, is not guaranteed or endorsed by the publisher.
